# Pyridine-N-Oxide Alkaloids from *Allium stipitatum* and Their Synthetic Disulfide Analogs as Potential Drug Candidates against *Mycobacterium tuberculosis*: A Molecular Docking, QSBAR, and ADMET Prediction Approach

**DOI:** 10.1155/2022/6261528

**Published:** 2022-10-07

**Authors:** Cedric Dzidzor Kodjo Amengor, Emmanuel Orman, Cynthia Amaning Danquah, Inemesit Okon Ben, Prince Danan Biniyam, Benjamin Kingsley Harley

**Affiliations:** ^1^Department of Pharmaceutical Chemistry, School of Pharmacy, University of Health and Allied Sciences, Ho, Ghana; ^2^Department of Pharmacology, Faculty of Pharmacy and Pharmaceutical Sciences, Kwame Nkrumah University of Science and Technology, Kumasi, Ghana; ^3^Department of Pharmacology, School of Pharmacy, University of Health and Allied Sciences, Ho, Ghana; ^4^Department of Pharmacognosy, School of Pharmacy, University of Health and Allied Sciences, Ho, Ghana

## Abstract

In this study, we consider pyridine-N-oxide alkaloids from *Allium stipitatum* and their synthetic disulfide analogs (PDAs) as candidates for next-generational antimycobacterial agents, in light of growing resistance to existing conventional therapies. *In silico* studies involving molecular docking simulations of 12 PDAs were carried out against 7 *Mycobacterium tuberculosis* target proteins (MTs) to determine their theoretical binding affinities. Compounds A3, A6, and B9 demonstrated stronger binding affinities on similar MTs. Molecular descriptors (MDs) describing structural and physicochemical properties of the compounds were also calculated using ChemDes, explored using Pearson's correlation analysis, and principal component analysis (PCA) in comparison with MDs from conventional antitubercular medicines. The PDAs possessed similar scores as isoniazid and pyrazinamide. The MDs were also used to conduct a quantitative structure-binding affinity relationship (QSBAR) study by building good fit and significant models through principal component regression (PCR) and partial least squares regression (PLSR). Leave-one-out cross-validation was adopted in the PLSR, resulting in good predictive models on all MTs (range of *R*^2^ = 0.7541‐0.8992; range of *Q*^2^ = 0.6183‐0.8162). Both PCR and PLSR models predicted the significant effects of ndonr, Hy, Mol wt, nhev, nring, ndb, Log P, W, Pol, ISIZ, TIAC, Getov, and UI on the binding of ligands to the MTs. *In silico* prediction of PDAs' ADMET profiles was conducted with QikProp utility. The ADMET profiles of the compounds were favorable. The outcome of the current study strengthens the significance of these compounds as promising lead candidates for the treatment of multidrug-resistant tuberculosis.

## 1. Introduction

Tuberculosis (TB) is an infectious disease with one of the leading causes of mortality in the world. Chemotherapy against this disease has remained a priority for the World Health Organization (WHO) [1], particularly because of the challenges associated with increased incidences of resistant strains and the toxicity of chemotherapeutic options available. This has led to the continuous search for novel, cost-effective antitubercular drugs with alternative mechanisms of action, and less susceptibility to resistance development [2].

As one of the most virulent bacteria due to its drug resistance, *Mycobacterium tuberculosis* had reportedly infected about 1.5 million people globally as of 2020 (including 214,000 HIV-positive people). One of its resistance mechanisms has been attributed to the very thick biological membrane combined with a waxy coating of mycolic acids [3, 4]. Furthermore, acquired resistance to selective antibiotic use against this organism through chromosomal mutations is also on the ascendency [5, 6]. Susceptible tuberculosis has been managed with a cocktail of drugs including the first-line TB drugs, isoniazid, ethambutol, pyrazinamide, and rifampicin through the directly observed therapy (DOT) approach. Previously, alternative therapies including the use of heavy metals and herbs were considered, but none of them has established any significant clinical success as far as a permanent solution is concerned [7]. The WHO reports that multi- and extensively drug-resistant TB presents a challenge in therapy. While multidrug-resistant TB is caused by strains that have developed resistance to at least rifampicin and isoniazid, extensively drug-resistant (XDR) TB is caused by strains, which in addition to being multidrug resistant, do not succumb to fluoroquinolones and to at least one of the second-line parenteral drugs, including, kanamycin, capreomycin, and amikacin. A more disturbing situation has been the emergence of *M. tuberculosis* strains which are resistant to all test antibiotics and have been described as total drug-resistant (TDR) TB [8].

As a result, there has been a pressing need for new cost-effective drugs with pleiotropic mechanisms of action to tackle this global pandemic. In recent times, there has been an increase in the use of herbal-based products as antitubercular drugs due to the myriad of therapeutic potentials from plants' secondary metabolites [9]. Medicinal plants remain a significant resource for the exploration of potential lead compounds with antitubercular activity (anti-TB). It is in this vein that in 2009, O'Donnell et al. explored the anti-TB potential of three (3) pyridine-N-oxide alkaloids isolated from the bulbs of *Allium stipitatum*, and Danquah et al. in 2016 further carried out synthetic optimization leading to a series of methyl disulfides which exhibited antitubercular activity through inhibition of *Mycobacteria* efflux pumps and biofilm [10]. There is however no sufficient information on their binding affinity or ligand-receptor molecular interactions. Following up on this trajectory, it was of interest to identify *Mycobacterium tuberculosis* targets for the pyridine-N-oxide alkaloids and their methyl disulfide analogs (PDAs) to facilitate drug optimization for improved efficacy and reduced cytotoxicity. The concept of reverse molecular docking and other biocomputational tools including quantitative structure-binding affinity relationship (QSBAR) modelling constitute cost-effective approaches to screen and validate *in vitro* bioassays or chemical optimization to facilitate the drug discovery process. Molecular docking is used to model the interactions between a drug molecule (referred to as a ligand) and a protein target at the atomic level, with an outcome expressed as ligand-binding efficiency [11]. The computational assessment also affords the opportunity to investigate and understand the binding properties of ligands through quantitative modelling of their structural features as against binding efficiencies on the targets: an approach termed as QSBAR [12]. A closely related and frequently adopted approach involves the quantitative modelling of the relationship between ligand structural features and outcomes from biological tests, and it is known as quantitative structure-activity relationship (QSAR) studies [13, 14]. The above-mentioned techniques have been widely useful in several drug discovery programmes. Recently, molecular docking has been used to predict the mode of action and binding affinity (docking scores) of 53 natural products known to have activity against TB based on existing essential antitubercular targets including ClpP1P2, DprE1, InhA, KasA, PanK, PknB, and Pks13 [15].

We herein report the assessment of the drug-like properties of the PDAs, evaluate their binding to known molecular targets (MTs) in comparison with MTs inhibitors and conventional anti-TBs, predict features of the compounds that contribute to their binding, and assess their absorption, distribution, metabolism, excretion, and toxicity (ADMET) profiles using in silico models. We propose that, understanding the physicochemical properties associated with ligand binding to molecular targets is essential to guiding the design and syntheses of more effective drug candidates to fight resistant TB infections.

## 2. Experimental

### 2.1. Dataset

**Figure 1:**
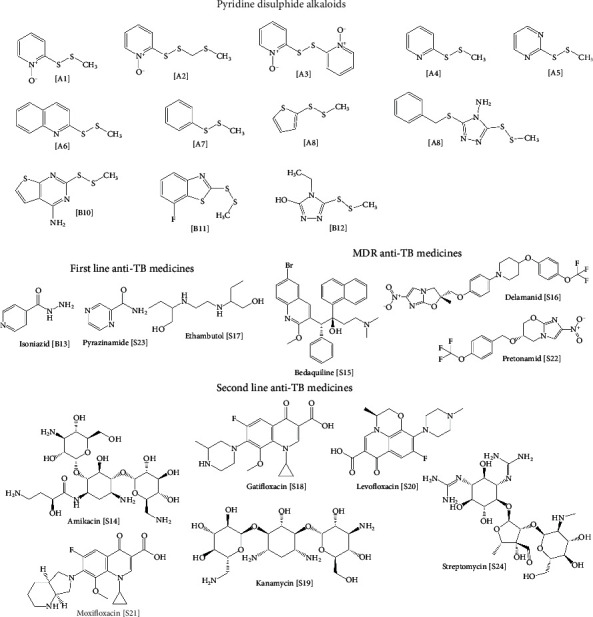
Structures of the conventional antitubercular medicines and PDSA used in the study. The PDSA included; **[A1]** 2-(((methylthio)methyl)disulfanyl)pyridine-1-oxide; **[A2]** 2,2′-disulfanediylbis (pyridine-1-oxide); **[A3]** 2-(methyldisulfanyl)pyridine-1-oxide; **[A4]** 2-(methyldisulfanyl)pyridine; **[A5]** 2-(methyldisulfanyl)pyrimidine; **[A6]** 2-(methyldisulfanyl)quinoline; **[A7]** 1-methyl-2-phenyldisulfane **[A8]** 2-(methyldisulfanyl)thiophene; **[B9]** 3-(benzylthio)-5-(methyldisulfanyl)-4H-1,2,4-triazole-4-amine; **[B10]** 2-(methyldisulfanyl) thieno [2,3-*d*]pyrimidin-4-amine; **[B11]** 7-fluoro-2-methyl(disulfanyl) benzo [*d*]thiazole; **[B12]** 4-ethyl-5-(methyldisulfanyl)-4H-1,2,4-triazol-3-ol.

The dataset used to conduct this study consisted of 24 drugs and drug-like compounds. Among them included three (3) first-line antitubercular medicines, six (6) second-line antitubercular medicines, three (3) multidrug-resistant antitubercular (MDR-TB) medicines, and twelve (12) PDAs from *Allium stipitatum* reported to have antitubercular activity against *Mycobacterium tuberculosis* H37Rv strain [10].  Figure 1 shows the chemical structures of the compounds included in the study.

### 2.2. Molecular Descriptors

20 1D and 2D molecular descriptors (MDs), which are quantitative estimates of the physicochemical and structural properties of the compounds, were calculated using the ChemDes integrated web-based platform [16]. These included 10 constitutional descriptors, 5 topology descriptors, 1 kappa descriptor, and 4 molecular property descriptors.  Table 1 details all the MDs considered. The MDs were then explored using correlation analysis and principal component analysis (PCA) to identify trends and patterns within the compounds (especially between the disulphide compounds and the conventional antitubercular medicines) and potentially classify the compounds using the unsupervised method based on their MDs (refer to Section 2.5). Prior to the PCA, the data was standardized to a mean of 0 and standard deviation of 1, and the principal components (PC), which were constructed as linear combinations of original variables to maximize the description of data variance, were selected based on their eigenvalues using Kaiser's rule. The PCA model with *f* principal components for a data matrix *X* could be presented as follows:

(1) $$\begin{array}{c} X={TP}^{T}+E, \end{array}$$

where *X* is a data matrix with *m* objects and *n* variables, *T* is the score matrix with dimensions (*m* × *f*), PT is a transposed matrix of loadings with dimensions (*f* × *n*), and *E* is a matrix of the residual variance (*m* × *n*) that is not explained by the first *f* principal components.

### 2.3. Computational Methods

#### 2.3.1. Computer System and Software

The molecular docking analysis was performed using PyRx software on a computer system with the following specifications; Processor (AMD A6-9225 Radeon R4, 5 compute cores 2C+3 G 2.60 GHz), Installed RAM (16.00 GB), System type (64-bit operating system, x64-based processor). Other softwares used included Biovia Discovery Studio 2021, ChemDraw Ultra software, and Open babel.

#### 2.3.2. Target Proteins and Their Preparation

Seven molecular targets of *M. tuberculosis* including caseinolytic peptidase (PClpP), decaprenylphosphoryl-*β*-D-ribose-2′-oxidase (DprE1), enoyl-acyl carrier protein (acp) reductase (InhA), 3-oxoacyl-[acyl-carrier-protein] synthase 1 (KasA), pantothenate kinase (PanK type 1), probable serine/threonine-protein kinase pknB, and polyketide synthase (Pks13) were selected as these are essential for bacterial survival, and their inhibition will affect the morphology and biochemistry of the mycobacterium [17]. The crystal structures and respective controls of ClpP1P2 (PDB ID: 4U0G), DprE1 (PDB ID: 6HEZ), InhA (PDB ID: 1ENY), KasA (PDB ID: 2WGE), PanK type 1 (PDB ID: 4BFT), PknB (PDB ID: 2FUM), and Pks13 (PDB ID: 5V3X) were retrieved from the RCSB Protein Data Bank (PDB) database (https://www.rcsb.org). Each control was bound to a pocket assumed to be the active site. The active site residues were obtained from PDBSUM database and were also confirmed in Biovia Discovery Studio 2021. To achieve good binding interactions between the molecular targets and the ligands, water molecules, ligand groups and all form of heteroatoms on the proteins were removed and saved in PDB format. The proteins were then transformed as macromolecules using PyRx.

#### 2.3.3. Ligand Preparation

The compounds which served as ligands for the docking studies included the PDAs, comprising the alkaloids isolated from *Allium stipitatum* [A1-A8] (*n* = 8) and the disulfide analogs [B9-B12] (*n* = 4) (Figure 1) synthesized based on the structural template of the natural alkaloids. The other compounds used included the first- and second-line conventional antitubercular medicines (*n* = 9) and MDR-TB medicines (*n* = 3). The structures of these compounds were drawn with ChemDraw Ultra software, converted to the Simplified Molecular Input Line Entry System (SMILES) format and uploaded into Open babel software to convert each compound to PDB format which was recognized by the PyRx software. The optimum conformations of the ligands at minimum energy were achieved and subsequently converted to Autodock ligand format (pdbqt) using PyRx software.

#### 2.3.4. Molecular Docking

The molecular docking interactions between the proteins and the ligands were computed to determine the binding affinities and to ascertain the possible binding sites using Autodock Vina of PyRx virtual screening software [18]. The Vina wizard uses a stochastic gradient optimization algorithm for predicting the binding affinities between ligands and molecular targets. In all cases, docking was performed in five technical runs. Validation of the docking protocols was performed by redocking the cocrystallized ligands bound to each protein (controls) into the binding pockets of each protein and compared to their bound conformations retrieved from PDB. The interaction types such as hydrogen bonding and hydrophobic interaction of docking output with the highest binding affinity were visualized using Biovia Discovery Studio Visualizer 2021. Additionally, the root-mean-square deviation (RMSD) was calculated using VMD software.

### 2.4. Quantitative Structure-Binding Affinity Relationship Study

**Figure 2:**
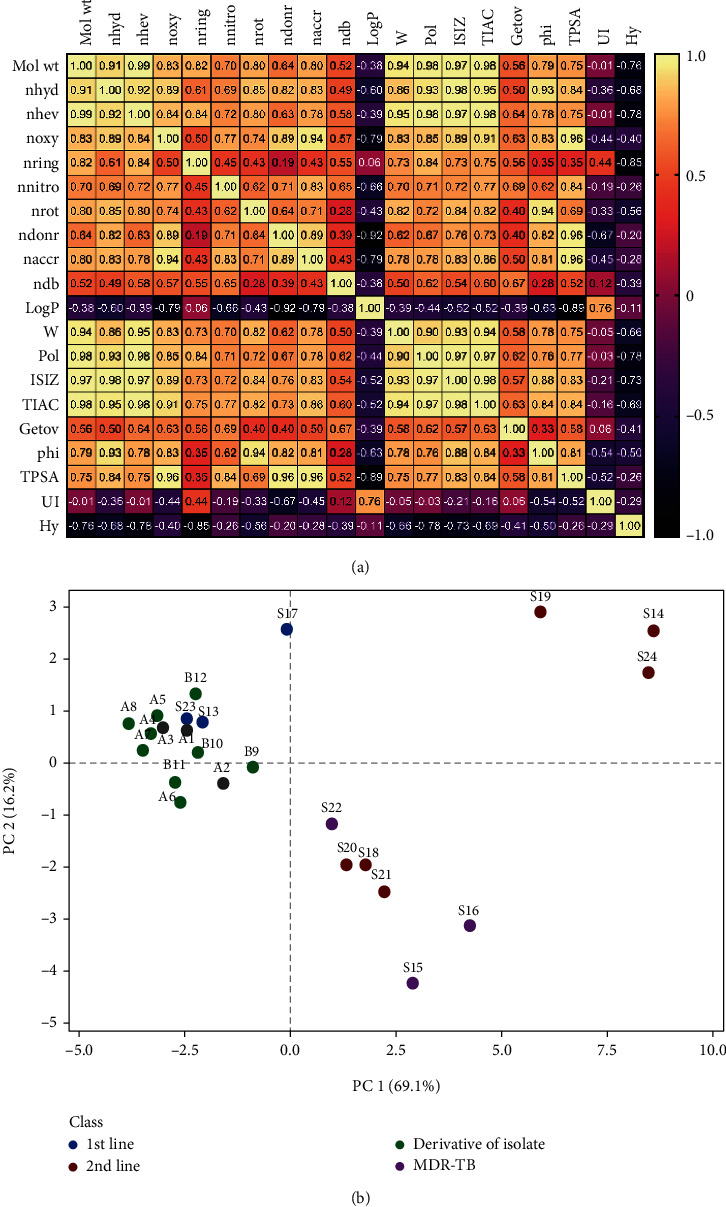
Correlation analysis (a) and principal component analysis (b) of the molecular descriptors of the ligands included in the study.

The QSBAR predictive modelling of the compounds was carried using principal component regression (PCR) and partial least squares regression (PLSR) analysis [17] from GraphPad Prism (version 9) and Minitab (version 18.1) softwares, respectively. In the PCR analysis, the appropriate number of PCs selected from the PCA on the MDs (Section 2.2) served as the independent predictors (*X*), and the dependent variables (*Y*) were the binding efficiencies of the ligands on the molecular targets (Figure 2). The multiple linear regression analysis part of the PCR was then performed according to

(2) $$\begin{array}{c} Y_{i}=\beta_{o}+\beta_{1}x_{i1}+\beta_{2}x_{i2}+\cdots +\beta_{k}x_{i.k}+\varepsilon_{i}, \end{array}$$

where *Y* is response variable, that is, the binding efficiency on each molecular target, *X*_1_, *X*_2_, ⋯*X*_*k*_ are the independent variables determined from the PCs, and *β*_o_, *β*_1_, *β*_2_, ⋯, *β*_*k*_ are coefficients or constants derived from the regression analysis using the PC scores. The coefficient estimates were the converted back to the scale of original variables using the linear combinations of variables defined for each PC.

The PLSR was also carried out to describe the relationship between the MDs, employed as the independent variables (*X*) and the ligand binding efficiencies, as the dependent variables (*Y*) using

(3) $$\begin{array}{c} y=X\times b+e, \end{array}$$

where *b* is the vector of the regression coefficients and *e* is the vector of the errors.

The PLSR models were developed for standardized data, and their complexity was estimated using leave-one-out cross-validation (LOO-CV) method. In this approach, the calibration process was repeated *m* times, with each time, treating the *i*th left-out object as the prediction object. The dependent variables for each left-out object were then calculated based on the model with two factors.

The descriptive and predictive power of the models was characterized using the multiple correlation coefficient (*R*), Fisher's ratio (*F*), predicted error sum of squares (PRESS), and *P* values. Additionally, the standardized coefficients of the independent descriptors in the PLSR provided information on the contribution of each MD to the target binding.

### 2.5. In Silico ADMET Prediction

Quantitative MDs for the purposes of ADMET prediction were calculated for the 12 PDAs using Maestro software (Maestro Version 12.5.139, MMshare Version 5.1.139, Release 2020-3, Platform Windows-x64) and QikProp utility (QikProp, Schrödinger, LLC, NY, 2017, Force Field of OPLS3e) [19]. The prediction of their interaction with cytochrome P450 (CYP3A4) was also carried out using the P450 Site of Metabolism tool, which is one of the tools of the Physics-Based ADME/Tox suite of the Maestro software. This tool identified the likely sites of metabolism based on Hammet and Taft-types rules and 3D spatial information. For the CYP3A4 isoform, the tool calculated only intrinsic reactivity and accessibility, labelled as overall site of metabolism (SOM) score. This was displayed as green circles, in which the radius was proportional to the score, where larger scores meant higher reactivity. The passive membrane permeability of the structures was calculated using the Membrane Permeability tool (ADME/Tox suite).

## 3. Results and Discussion

### 3.1. Exploring Molecular Descriptors of Ligands

The MDs selected were classified as constitutional, topology, kappa, and molecular property descriptors (Table 1). Constitutional descriptors relate to information on the chemical composition of the compounds without regard to their molecular geometry or atomic connectivity [20]. Topological descriptors consider the internal arrangement of atoms in the compounds and encodes for information on molecular size, shape, branching, and the presence of heteroatoms and multiple bonds [21]. The kappa descriptor Kier molecular flexibility index which is a measure of the flexibility of the compounds [20] was also included. The molecular property descriptors describe physicochemical and biological properties as well as some molecular characteristics of the compounds. Together, these descriptors encode for significant chemical information about different aspects of the compounds considered in the study [20]. A Pearson's correlation analysis showed a high degree of collinearity among the MDs. To adequately explore these data with an unsupervised approach, while reducing the collinearity effect, PCA was used. By using the Kaiser's rule, the first three PCs were selected to cumulatively describe 92.4% of the variation in the data. PC1 accounted for most of the variations related to constitutional, topology and kappa descriptors. PC2 on the other hand accounted for the variation in the molecular property descriptors, and PC3 accounted for variations in specific descriptors like ndb and Getov.

A cluster of scores of the pyridine disulfide alkaloids (A1-B12) with three first-line anti-TB medicines, S13 and S23 was observed, and these were characterized by their relatively low Log*P*s (range: -0.43 and 3.68), UIs (range: 0.00–3.70), and Hys (range: (-)2.85–(-)1.75). The second-line and MDR anti-TB medicines on the other hand were clustered around different spatial regions characterized by relatively higher number of rings [nring] (range: 3–5) than the first-line anti-TB medicines for one of the clusters and a second cluster characterized by high correlation of all other descriptors. This second other cluster comprised mainly the subsection of the second-line medicines. In effect, the pyridine disulfide alkaloids being investigated were observed to possess structural and physicochemical properties (that is, Log*P*, Hy, and UI) like the first-line anti-TB medicines, including isoniazid (S13) and pyrazinamide (S23).

### 3.2. Molecular Docking Studies

#### 3.2.1. Docking Validation

**Figure 3:**
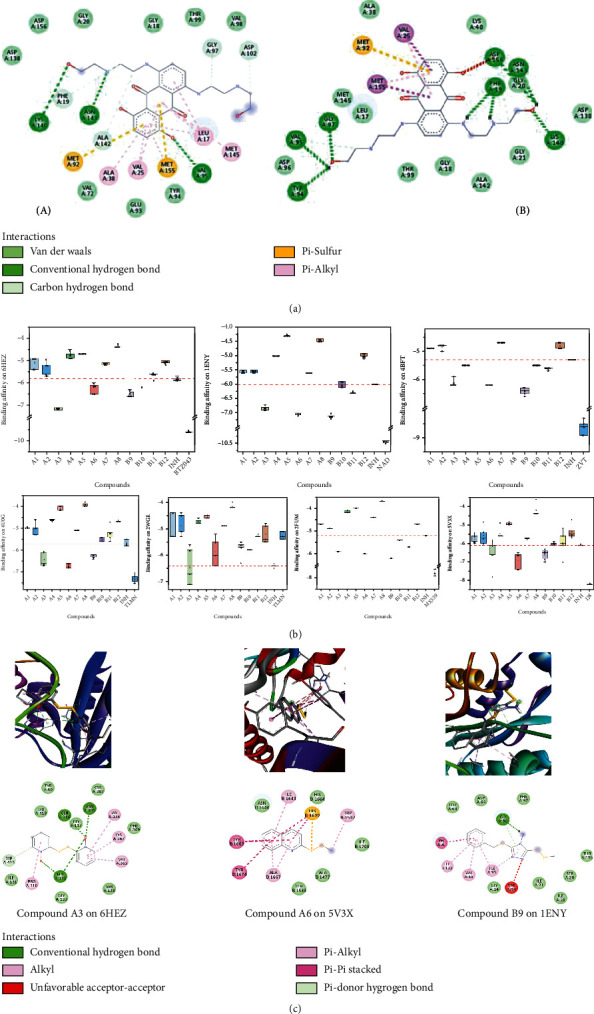
Summary of results from molecular docking studies. (a) Comparison of the docking outputs (B) with the natives (A) showing comparable binding pocket interactions. (b) Binding energies observed for the PDAs on the molecular targets considered. These were compared with that of the respective inhibitors (BTZ043, NAD, ZVT, TLMN, MX539, and I28) and isoniazid (INH). (c) 3D and 2D binding models of most effective ligands on selected molecular targets. The figure also shows the different amino acid units present at the respective binding sites.

To validate the docking methods, the cocrystallized ligands bound to the respective molecular targets were redocked into their binding pockets. Comparison of the docking outputs (b) with the natives (a) revealed comparable binding pocket interactions. An example is illustrated in Figure 3(a). The interactions of the inhibitor, MIX 539 with Lys 140, ASN 143, Val 95, Phe 19, Gly 97, Tyr 94, Asp 156, Met 92, Met 155, Val 25, Ala 38, Leu 17, Met 145, Asp 138, Gly 18, Ala 142, Thr 99, and Gly 20, were common in both conformations of PknB (PBD ID 2FUM) (RMSD = 0.695 Å). Eight (8) conventional hydrogen bonds (distances between 1.93 and 2.55 Å), two *π* − *σ* bond, one *π*-sulphur (4.41 Å), 2 *π*-alkyl bonds (*π*-interaction with Val 25 and Met 155 at distances of 4.87 and 4.67 Å, respectively), two C-H bond with Val 95 and Asp 156 amino acid residues at distances of 3.28 and 3.36 Å, respectively, eleven Van der Waals, and one unfavourable acceptor and acceptor bond (2.71 Å between the aromatic C-O and ASP 156 amino acid residue) were observed. The binding scores from the validation docking were between -7.6 and -7.9 kcal/mol, and this was consistent with that obtained by Baptista et al. (that is, -7.7 kcal/mol) [22].

#### 3.2.2. Docking of the Pyridine Disulfide Analogs

The molecular docking simulation was performed to confirm the *in vitro* antitubercular activity of the PDAs reported in literature [10]. They were docked against the mycobacterial targets ClpP1P2 (PDB ID: 4U0G), DprE1 (PDB ID: 6HEZ), InhA (PDB ID: 1ENY), KasA (PDB ID: 2WGE), PanK type 1 (PDB ID: 4BFT), PknB (PDB ID: 2FUM), and Pks13 (PDB ID: 5V3X) to identify possible binding interactions between the proteins and the alkaloids. ClpP1P2 carries out the energy-dependent degradation of abnormal proteins within the cells during *in vitro* growth and infection. DprE1 is a decaprenylphosphoryl-d-ribose oxidase involved in the biosynthesis of decaprenylphosphoryl-D-arabinose, an essential component of the mycobacterial cell wall and thus is essential for cell growth and survival. InhA is a known target of isoniazid, essential for the synthesis of mycolic acids. KasA is one of the key enzymes responsible for the elongation of C16–26 fatty acyl primers in the FAS-II system for mycolic acid production of *M. tuberculosis*. Pantothenate kinase (PanK) is a key enzyme in the universal biosynthesis of the essential cofactor CoA. PknB is a very well-characterized mycobacterial serine/threonine-protein kinase involved in cell growth control. Pks13 is an essential enzyme that forms mycolic acids, required for the cell wall of *Mycobacterium tuberculosis* [23].

For all docking simulations, nine poses were obtained and evaluated. The binding energies observed ranged from -4 to -7.1 kcal/mol, -4.2 to -6.6 kcal/mol, -4.2 to -7.2 kcal/mol, -4.0 to -7.1 kcal/mol, -4.0 to -6.6 kcal/mol, -3.7 to -6.2 kcal/mol, and -4.4 to -6.8 kcal/mol against ClpP1P2 (PDB ID: 4U0G), DprE1 (PDB ID: 6HEZ), InhA (PDB ID: 1ENY), KasA (PDB ID: 2WGE), PanK type 1 (PDB ID: 4BFT), PknB (PDB ID: 2FUM), and Pks13 (PDB ID: 5V3X), respectively (Figure 3(b)). Compounds A1, A2, A5, A7, A8, and B12 possessed relatively poor binding affinities on all the seven molecular targets considered in comparison to isoniazid (INH). Compounds A3 and A6 demonstrated similar-to-better binding affinities on all molecular targets whiles compound B9 also had similar-to-better binding affinities on 6 of the targets. Compound A4 had similar affinity to only PanK type 1 (PDB ID: 4BFT). The binding of compounds A3, A6, and B9 are illustrated in Figure 3(c). The molecular target PanK type 1 (PDB ID: 4BFT) was susceptible to a greater number of the ligands (*n* = 6/12), followed by DprE1 (PDB ID: 6HEZ), PknB (PDB ID: 2FUM), InhA (PDB ID: 1ENY), and Pks13 (PDB ID: 5V3X) (*n* = 5/12). The target KasA (PDB ID: 2WGE) was the least susceptible, with only 2 ligands showing appreciable binding affinities. Also, binding affinities on InhA (PDB ID: 1ENY) were shown to be generally higher (-6.1 to -7.2 kcal/mol) as compared to affinities on other targets (Figure 3(b)).

### 3.3. Quantitative Structure-Binding Affinity Relationship Studies

**Figure 4:**
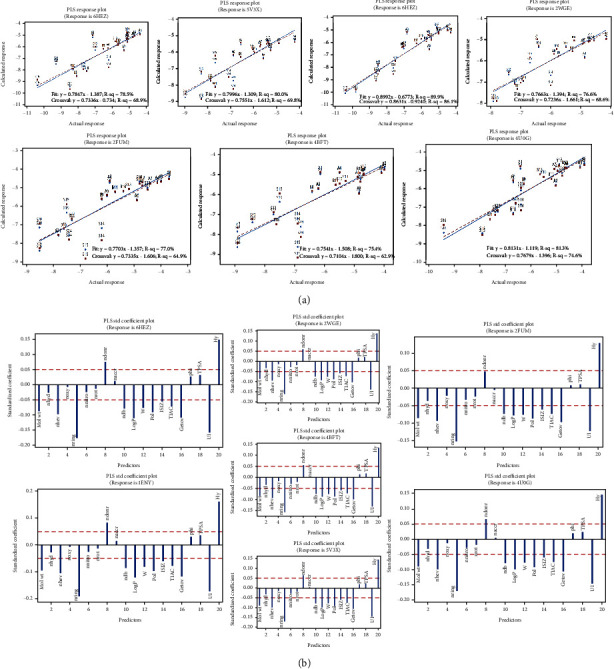
Outcomes from partial least regression analysis in the quantitative structure-binding affinity relationship study. (a) PLS regression fits with cross-validation fits showing good prediction abilities of the models developed for each molecular target. (b) Standardized coefficients of the MDs in the PLS analysis. Red horizontal line is an imaginary line drawn to identify MDs with significant coefficients to the binding on the respective targets.

As previously indicated, PCR and PLSR models were used to study the relationship between the MDs and the binding affinities on the molecular targets. In the PCR, PC1-PC3 were selected based on their eigenvalues to build the regression models that could predict the binding efficiencies (*y*) on the molecular targets. The PCR models were developed by least squares and were significant on each target binding site considered (Table S1). The outcomes show that on all the targets, constitutional descriptors including Mol wt, nhev, nring, and ndonr significantly contributed to ligand-binding efficiencies (Table S2). Topology descriptors, including, W, Pol, ISIZ, and TIAC also had significant impact on the binding efficiencies on all targets. UI and Hy, which are molecular property descriptors, also significantly influenced binding on all sites. The MDs, nrot, and phi did not have any significant impact on the binding on these targets. Others, including noxy, naccr, and TPSA, also did not generally influence binding of the ligands. Tables S1-S3 details the outcomes of the PCR regression. In the PLSR, the best models were selected based on high values of *R*^2^, predicted *R*^2^ from cross-validation (*Q*^2^), *F* values, and low values of PRESS (Tables S4-S6). Models with *R*^2^ > 0.6 and *Q*^2^ > 0.5, with preferably a difference between them ranging between 0.2 and 0.3 were considered suitable for the prediction of the relationship between the MDs and the binding efficiencies on the molecular targets [24]. Each of the models developed were consistent with the assertion and were found to be significant (*P* < 0.0001). Based on the *X*-variance, *R*^2^, predicted *R*^2^ (*Q*^2^), and PRESS, 2 latent variables were selected to describe the relationship between the dependent and independent datasets. The 2 latent variables cumulatively explained 85% of the variation in the MDs. Generally, the points in the response plots followed linear patterns, indicating that the models fitted the data well. The plots also did not show large differences between the fitted and cross-validated fitted responses (Figure 4(a)). From the analysis, the binding efficiencies on all targets considered were largely dependent on the positive standardized coefficients of ndonr and Hy and on negative coefficients of Mol wt, nhev, nring, ndb, Log P, W, Pol, ISIZ, TIAC, Getov, and UI (Figure 4(b)). Effectively, increasing the number of hydrogen bond donors and hydrophilicity of the ligands tends to increase the binding efficiencies on all targets. It was evident that first- and second-line anti-TB medicines had higher numbers of hydrogen bond donors as compared to the PDAs, where they were virtually absent. Future structural modifications could consider increasing this feature to further enhance binding efficiency. On the other hand, the hydrophilicity indices of the PDAs were comparatively higher than that of the first- and second-line anti-TB medicines. With regard to the MDs with negative coefficients, decreasing their corresponding values and effects is predicted to enhance the binding efficiencies on the targets. Hence, structures with relatively lower molecular weights, lower numbers of heavy atoms and rings, as well as lower LogPs, polarity indices, total information indices on molecular sizes and atomic compositions, among others would preferably produce better binding efficiencies than that observed. Thus, future modifications on the structures of the PDAs could consider these options.

### 3.4. In Silico ADMET Prediction

#### 3.4.1. Drug-Likeness Prediction

The drug-likeness properties of ligands relate to their aqueous solubility and gut blood barrier permeability, which determines the first step of oral bioavailability. The assessment of the compounds was carried out using the Lipinski's Rule of 5 (Ro5) and Jorgensen's Rule of 3 (Ro3) [25–27]. These are guidelines established to determine the number of violations of Lipinski's rule of five, including molecular weight (Mol wt < 500), predicted octanol/water partition coefficient (*QP*log*P*o/w = −2.0–6.5), number of hydrogen bond donors (nHBD ≤ 5), number of hydrogen bond acceptors (nHBA ≤ 10), and topological surface area (TPSA < 40 Å^2^), and violations of Jorgensen's rule of three, which are predicted aqueous solubility (QPlogS > −5.7), predicted apparent Caco-2 cell, a model for the gut-blood barrier (QPPCaco: <25: poor, >500: great), and number of primary metabolites (#metab < 7). Compounds with fewer (and preferably no) violations of these rules are considered drug-like and are more likely to be orally available [26]. It was observed that none of the compounds A1-B12 violated both rules (Table 2). The results therefore suggest that the compounds possessed drug-like features, to be considered for future drug developments.

#### 3.4.2. Bioavailability Prediction

Oral bioavailability is an essential molecular descriptor in drug design of a compound for the processes of absorption and liver first-pass metabolism [28]. Absorption, however, depends on the solubility and permeability of the compound across the cell membranes as well as interactions with transporters and metabolizing enzymes in the gastrointestinal tract. The computed molecular descriptors used to assess oral absorption are the predicted aqueous solubility (QPlogS), the predicted qualitative human oral absorption (%PHOA), and predicted apparent Caco-2 cell, a model for the gut-blood barrier permeability in compliance to Jorgensen's famous Ro3 (QPPCaco). The compounds were predicted to have high human oral absorption (85-100%), with 83% of them having 100% score, and this correlates with their Caco-2 cell line permeability of >500 nm/sec. Caco-2 cell lines are used as an intestinal permeability model and contain enterocytes lining the intestine which are characterized by transporters such as p-glycoprotein. This molecular descriptor is used to mimic human intestinal mucosal absorption characteristics and hence used to predict oral absorption of drugs in humans making it a rapid *in vitro* screening tool in support of drug discovery within the pharmaceutical industry, as well as biomolecular dynamics including intestinal absorption, transport, and metabolism. This suggests that the compounds also had great predicted propensity to be substrates of P-gp efflux transporters indicating that they could undergo passive absorption via P-gp.

#### 3.4.3. Prediction of Blood–Brain Barrier (BBB) Penetration

When drugs are too hydrophilic, they do not cross the blood brain barrier [29]. The blood/brain partition coefficients (QPlogBB) were computed and used as a predictor for access to the central nervous system (CNS). The predicted CNS activity was computed on a −2 (inactive) to +2 (active) scale and showed that only 25% of the compounds (A4, A6, and B11) could be very active in the CNS (predicted CNS activity > 1, more positive CNS effect). The positive CNS effect meant that these compounds can possibly produce therapeutic or toxic CNS-related effects. The calculated apparent MDCK cell permeability (QPPMDCK) is considered a good mimic for the BBB (for passive transport). Its estimates showed that the compounds with MDCK cell permeabilities falling within the recommended range of 25–500 nm s^−1^ for 95% of known drugs could undergo passive absorption.

#### 3.4.4. Prediction of Skin Permeability

This MD is important for drugs formulated for topical application. The distribution of computed skin permeability parameter (QPlogKp) showed that all the compounds fall within the recommended range for 95% of known drugs.

#### 3.4.5. Prediction of Human Serum Albumin Binding

The efficacy of a drug could be affected by the degree to which it binds to the proteins within blood plasma. It is noteworthy that binding of drugs to plasma proteins (like human serum albumin, lipoproteins, glycoproteins, and globulins) greatly reduces the quantity of the drug in general blood circulation, and hence, the less bound a drug is, the more efficiently it can traverse cell membranes or diffuse [30, 31]. Human serum albumin is the most abundant plasma protein, and it is known for its unique binding capacity to drugs. It serves as a main cache to determine the active concentration influencing the duration of action thereby affecting the ADME properties; hence, the predicted plasma-protein binding has been estimated using the binding to human serum albumin; the QPlogKhsa parameter (recommended range is −1.5 to 1.5 for 95% of known drugs). Our estimation revealed that all the compounds are compliant to this parameter, indicating that most of the compounds have a high probability of circulating freely within the blood stream and hence have access to the target site.

#### 3.4.6. Prediction of Blockage of Human Ether-a-Go-Go-Related Gene Potassium (HERG K+) Channel

**Figure 5:**
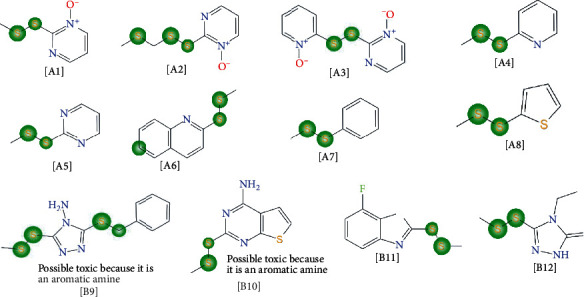
Predicted sites of metabolism reactivity of disulfide analogs. CYP3A4 reactivity site indicated by green circle.

Human ether-a-go-go-related gene (HERG) encodes a potassium ion (K+) channel that is implicated in the fatal arrhythmia known as torsade de pointes or the long QT syndrome [32]. The HERG K+ channel which is best known for its contribution to the electrical activity of the heart coordinates heart's beating and appears to be the molecular target responsible for cardiac toxicity of a wide range of therapeutic drugs. Thus, HERG K+ channel blockers are potentially toxic, and the predicted IC_50_ values often provide reasonable predictions for cardiac toxicity of drugs in the early stages of drug discovery [33]. Therefore, improving the ability to avoid undesirable hERG activity in the early stage of drug discovery is significant. Prediction of this phenomenon will improve the safety of therapy and enhance the process of drug development. In this work, the estimated or predicted IC_50_ values for blockage of this channel have been used to model the process in silico. Except for B9 and B10 which are the likely toxic candidate to cause an unwanted blockage of the potassium ion channel of the human ether-a-go-go-related gene (hERG) (Figure 5), the compounds met the requirement for this molecular descriptor. This virtual screening suggests that the compounds cannot induce hERG-related cardiotoxicity [34, 35].

#### 3.4.7. Prediction of Primary Metabolism

An estimated number of possible metabolic reactions were also predicted by QikProp and used to determine whether the molecules can easily gain access to the target site after entering the blood stream. The average recommended estimated number of possible metabolic reactions was between 1 and 8. From Table 2, the maximum metabolic steps was 3 for compound A3 whilst the rest fell within the range of 0-2. The results presented in Figure 5 show the various sites on the structures with the various sites of metabolism (SOM) accessible by the human cytochrome P450 3A4 (CYP3A4), an important enzyme mainly in the liver and responsible for the metabolism of more than 80% of medicines. This enzyme contains heme, and it catalyzes mainly 40-45% of phase 1 metabolic reactions such as hydroxylation, oxidation, and dealkylation [36].

#### 3.4.8. Permeability Prediction

The permeability of the compounds was estimated from calculations based on a physical model assumption dominated by the free energy of desolvation and change of state (neutralization and tautomerization) on passing into the membrane. This tool was modelled as a low-dielectric continuum and water as a high-dielectric continuum. The model was also optimized to reproduce RRCK permeability assay. Table 2 also shows the total free energy penalty state change of the structures and the states for which the structures could enter the membrane, moving from high dielectric region to low dielectric region and the logarithm of the RRCK permeability, an optimized property to reproduce RRCK permeability assay. The calculated “Log perm RRCK” values of bigger value (less negative number) mean more permeable, and for “Membrane dG Insert”, a more negative (or less positive) value means more permeable. This could mean that compounds A4, A7, A8, and B11 are predicted to be more permeable of all the listed structures as these have less positive values of the evaluated structures.

## 4. Conclusion

The pyridine disulfide alkaloids and their synthetic analogs earlier on reported to possess in vitro antitubercular activity have in this study been shown to possess structural features and chemical information similar to isoniazid and pyrazinamide. Compounds A3, A6, and B9 showed favorable binding efficiencies on all the molecular targets considered. Ligand binding was thought to be better when the number of hydrogen bond donors (ndonr) and hydrophilicity index (Hy) increased. Similarly, the binding also became better when the following feature effects on the compounds were reduced: Mol wt, nhev, nring, ndb, Log P, W, Pol, ISIZ, TIAC, Getov, and UI. The compounds also possessed favorable predicted ADMET profiles. With this information, it is possible to further design synthetic analogs of the PDAs with good binding effects and possibly target multi- and extended-drug-resistant strains of *Mycobacteria tuberculosis* bacteria.

## Figures and Tables

**Table 1:** Description of molecular descriptors adopted for the study.

Sr. no.	Group	Molecular descriptor	Description	Summary statistics (range)
1	Constitutional	Mol wt	Molecular weight	123.1–585.6
2		nhyd	Count of hydrogen atoms	5–43
3		nhev	Count of heavy atoms	8–40
4		noxy	Count of oxygen atoms	0–13
5		nring	Number of rings	0–5
6		nnitro	Count of nitrogen atoms	0–7
7		nrot	Number of rotatable bonds	1–11
8		ndonr	Number of hydrogen bond donors	0–13
9		naccr	Number of hydrogen bond acceptors	2–17
10		ndb	Number of double bonds	0–3
11	Topology	W	Weiner index	67–6255
12		Pol	Polarity number	5–76
13		ISIZ	Total information index on molecular size	53.3–529.1
14		TIAC	Total information index on atomic composition	21.43–138
15		Getov	Geometric topological index by Narumi	1.54–4.09
16	Kappa	phi	Kier molecular flexibility index	1.40–11.95
17	Molecular property	Log*P*	Log *P* value based on the Crippen method	(-)8.42–(+)7.13
18		TPSA	Topological polarity surface area	0–336.4
19		UI	Unsaturation index	0–4.86
20		Hy	Hydrophilic index	(-)4.51–(-)1.99

**Table 2:** ADME/Tox predicted values of the disulfide alkaloids.

Cpd ID	Drug-likeness (no. of violations)		Oral bioavailability prediction			Cardiotoxicity prediction	Blood-brain barrier (BBB) penetration			Skin permeability	Protein binding	Primary metabolism	Passive membrane permeability	
	Ro5	Ro3	QPlogS	%PHOA	QPPCaco (nm/sec)	QPlogHERG	QPlogBB	CNS	QPPMDCK (nm/sec)	QPlogKp	QPlogKhsa	#metab	Membrane dG insert (kcal/Mol)	Log perm RRCK (cm/s)
A1	0	0	-1.78	100	3034.94	-3.60	0.21	1	4518.15	-1.84	-0.51	1	6.229630	-4.359774
A2	0	0	-2.58	100	3038.80	-4.23	0.15	1	7490.62	-1.64	-0.36	1	5.336709	-4.376275
A3	0	0	-2.83	100	1523.45	-4.60	-0.16	0	1873.12	-1.80	-0.17	3	10.959890	-4.673919
A4	0	0	-2.09	100	8470.62	-3.85	0.59	2	10000	-0.83	-0.27	1	2.591768	-4.204556
A5	0	0	-1.61	100	5091.54	-3.68	0.41	1	8584.47	-1.37	-0.57	2	6.893223	-4.376285
A6	0	0	-3.61	100	8544.57	-4.62	0.59	2	10000	-0.56	0.14	0	4.415087	-4.375935
A7	0	0	-2.73	100	9906.04	-3.89	-0.09	0	10000	-0.64	-0.11	0	-0.264257	-4.094258
A8	0	0	-2.90	100	9906.04	-3.55	0.15	1	10000	-0.83	-0.06	1	1.752616	-4.147198
B9	0	0	-2.71	85	303.06	-5.98	-0.05	1	626.22	-4.03	-0.12	1	4.308330	-4.446987
B10	0	0	-2.86	100	1714.01	-3.86	0.05	1	5095.63	-2.31	-0.29	1	11.416972	-4.640013
B11	0	0	-3.53	100	6345.49	-4.15	0.67	2	10000	-1.15	-0.04	1	2.017210	-4.245295
B12	0	0	-2.09	90	953.75	-3.17	-0.33	0	1135.252	-3.188	-0.45	0	6.398424	-4.404494

QPlogS: predicted aqueous solubility (-6.5-0.5); %PHOA: predicted human oral absorption on 0 to 100% scale (>80%: great, <25% is poor); QPPCaco: predicted apparent Caco-2 cell (a model for the gut-blood barrier) permeability in nm/sec (<25: poor, >500: great); QPlogHERG: predicted IC_50_ value for blockage of HERG K+ channels (concern below -5); QPlogBB: predicted brain/blood partition coefficient (-3 to 12.0); CNS: predicted central nervous system activity on a –2 (inactive) to +2 (active) scale; QPPMDCK: predicted apparent MDCK cell permeability in nm/sec. MDCK cells are considered to be a good mimic for the blood-brain barrier (<25: poor, >500: great). QikProp predictions of Caco and MDCK are for nonactive transport; QPlogKp: predicted skin permeability, log Kp. (-8.0-1.0). QPlogKhsa: prediction of binding to human serum albumin (-1.5 to 1.5); #metab: number of likely metabolic reactions (1-8).

## Data Availability

Dataset for this study can be found at the Department of Pharmaceutical Chemistry, University of Health and Allied Sciences, and Department of Pharmacology, Kwame Nkrumah University of Science and Technology.

## Additional Points

*Key Findings*. (i) The pyridine disulfide alkaloids and their synthetic analogs earlier on reported to possess in-vitro antitubercular activity have been shown to possess structural information comparable to isoniazid and pyrazinamide in the QSBAR study. (ii) Compounds A3, A6, and B9 showed favorable binding efficiencies on all the molecular targets with increased number of hydrogen bond donors (ndonr) and hydrophilicity index (Hy). (iii) The compounds also possessed favorable ADMET profiles upon prediction.

## Supplementary Materials

Supplementary Materials Table S1: summary of ANOVA and fit model statistics from principal component regression (PCR). Table S2: summary of the significance of molecular descriptors to respective molecular targets in the PCR analysis. Table S3: estimated coefficients of molecular descriptors on the respective molecular targets in the PCR analysis. Table S4: summary of ANOVA and fit model statistics from partial least squares regression (PLSR) analysis. Table S5: summary of the significance of molecular descriptors to respective molecular targets in the PLSR analysis. Table S6: standardized coefficients of molecular descriptors on the respective molecular targets in the PLSR analysis.
