# Supplementary material for: Pyridine-N-Oxide Alkaloids from Allium stipitatum and Their Synthetic Disulfide Analogs as Potential Drug Candidates against Mycobacterium tuberculosis: A Molecular Docking, QSBAR, and ADMET Prediction Approach
Source: Biomed Res Int. 2022 Oct 7;2022:6261528. doi: 10.1155/2022/6261528 (PMC9568345; doi:10.1155/2022/6261528)
Supplement: Supplementary Materials — Table S1: summary of ANOVA and fit model statistics from principal component regression (PCR). Table S2: summary of the significance of molecular descriptors to respective molecular targets in the PCR analysis. Table S3: estimated coefficients of molecular descriptors on the respective molecular targets in the PCR analysis. Table S4: summary of ANOVA and fit model statistics from partial least squares regression (PLSR) analysis. Table S5: summary of the significance of molecular descriptors to respective molecular targets in the PLSR analysis. Table S6: standardized coefficients of molecular descriptors on the respective molecular targets in the PLSR analysis. [file 6261528.f1.docx]

### Pyridine-N oxide alkaloids from Allium stipitatum and their synthetic disulfide analogues as potential drug candidates against Mycobacterium tuberculosis: A Molecular Docking, QSBAR and ADMET Prediction Approach

^1^Cedric Dzidzor Kodjo Amengor,^1^Emmanuel Orman, ^2^Cynthia Amaning Danquah, ^1^Prince Danan Biniyam, ^3^Inemisit Okon Ben, ^4^Benjamin Kingsley Harley.

^1^ Department of Pharmaceutical Chemistry, School of Pharmacy, University of Health and Allied Sciences, Ho-Ghana.

^2^ Department of Pharmacology, Faculty of Pharmacy and Pharmaceutical Sciences, Kwame Nkrumah University of Science and Technology, Kumasi-Ghana.

^3^ Department of Pharmacology, School of Pharmacy, University of Health and Allied Sciences, Ho. Ghana.

^4^Department of Pharmacognosy, School of Pharmacy, University of Health and Allied Sciences, Ho. Ghana.

**Correspondence Author**

Cedric Dzidzor Kodjo Amengor (Ph.D)

Contact: Email: [camengor@uhas.edu.gh](mailto:camengor@uhas.edu.gh).

Tel: +233 (0) 246-456-764.

**PRINCIPAL COMPONENT REGRESSION ANALYSIS RESULTS**

Table S1 – Summary of ANOVA and Fit Model Statistics from Principal Component Regression (PCR)

| **Molecular target** | **ANOVA Analysis** | | **Goodness of Fit** | | |
| --- | --- | --- | --- | --- | --- |
|  | **F-value** | **p-value** | **R^2^** | **Sum of Squares** | **Sy.x** |
| 6HEZ | 23.86 | <0.0001 | 0.7816 | 13.09 | 0.8091 |
| 4BFT | 21.53 | <0.0001 | 0.7636 | 11.79 | 0.7679 |
| 2FUM | 24.85 | <0.0001 | 0.7885 | 10.45 | 0.7229 |
| 1ENY | 59.73 | <0.0001 | 0.8996 | 6.556 | 0.5726 |
| 5V3X | 25.35 | <0.0001 | 0.7918 | 7.388 | 0.6078 |
| 4UOG | 28.68 | <0.0001 | 0.8114 | 7.764 | 0.623 |
| 2WGE | 25.3 | <0.0001 | 0.7915 | 5.823 | 0.5396 |

Table S2 – Summary of the significance of molecular descriptors to respective molecular targets in the PCR Analysis

| **MD** | **6HEZ** | **4BFT** | **2FUM** | **1ENY** | **5V3X** | **4U0G** | **2WGE** |
| --- | --- | --- | --- | --- | --- | --- | --- |
| Mol wt | **** | **** | **** | **** | **** | **** | **** |
| nhyd | ns | ns | ns | ** | * | * | ns |
| nhev | **** | **** | **** | **** | **** | **** | **** |
| noxy | ns | ns | * | ns | ns | ns | ns |
| nring | **** | **** | **** | **** | **** | **** | **** |
| nnitro | ns | * | * | ns | ns | ns | * |
| nrot | ns | ns | ns | ns | ns | ns | ns |
| ndonr | ** | * | * | **** | ** | ** | * |
| naccr | ns | ns | ns | * | ns | ns | ns |
| ndb | * | ** | ** | * | ns | ns | ** |
| LogP | ** | ns | ns | **** | ** | ** | ns |
| W | **** | **** | **** | **** | **** | **** | **** |
| Pol | **** | **** | **** | **** | **** | **** | **** |
| ISIZ | *** | ** | *** | **** | **** | **** | ** |
| TIAC | **** | **** | **** | **** | **** | **** | **** |
| Getov | * | ** | ** | * | ns | * | ** |
| phi | ns | ns | ns | ns | ns | ns | ns |
| TPSA | ns | ns | ns | ** | ns | ns | ns |
| UI | **** | *** | *** | **** | *** | **** | **** |
| Hy | *** | ** | ** | **** | *** | **** | ** |

* *p* < 0.05, ***p* < 0.01, ****p* < 0.001 and *****p* < 0.0001

Table S3 – Estimated Coefficients of molecular descriptors on the respective molecular targets in the PCR analysis

|  | **6HEZ** | **4BFT** | **2FUM** | **1ENY** | **5V3X** | **4U0G** | **2WGE** |
| --- | --- | --- | --- | --- | --- | --- | --- |
| Intercept | -2.6520 | -2.7710 | -2.5620 | -2.5710 | -3.6670 | -2.8650 | -3.3840 |
| Mol wt | -0.0009 | -0.0007 | -0.0007 | -0.0011 | -0.0007 | -0.0008 | -0.0005 |
| nhyd | -0.0029 | -0.0024 | -0.0024 | -0.0056 | -0.0037 | -0.0042 | -0.0010 |
| nhev | -0.0133 | -0.0113 | -0.0111 | -0.0158 | -0.0106 | -0.0116 | -0.0083 |
| noxy | -0.0027 | -0.0078 | -0.0103 | 0.0014 | -0.0028 | -0.0027 | -0.0062 |
| nring | -0.1870 | -0.1544 | -0.1486 | -0.2086 | -0.1361 | -0.1488 | -0.1204 |
| nnitro | -0.0427 | -0.0583 | -0.0676 | -0.0107 | -0.0218 | -0.0219 | -0.0530 |
| nrot | 0.0010 | 0.0049 | 0.0067 | -0.0170 | -0.0087 | -0.0103 | 0.0096 |
| ndonr | 0.0269 | 0.0171 | 0.0139 | 0.0322 | 0.0176 | 0.0195 | 0.0140 |
| naccr | 0.0060 | 0.0000 | -0.0025 | 0.0097 | 0.0031 | 0.0036 | 0.0003 |
| ndb | -0.2166 | -0.2274 | -0.2429 | -0.1411 | -0.1229 | -0.1294 | -0.2017 |
| LogP | -0.0392 | -0.0233 | -0.0178 | -0.0551 | -0.0300 | -0.0335 | -0.0164 |
| W | -0.0001 | -0.0001 | -0.0001 | -0.0001 | -0.0001 | -0.0001 | 0.0000 |
| Pol | -0.0061 | -0.0054 | -0.0053 | -0.0070 | -0.0048 | -0.0052 | -0.0040 |
| ISIZ | -0.0006 | -0.0005 | -0.0005 | -0.0008 | -0.0005 | -0.0006 | -0.0003 |
| TIAC | -0.0029 | -0.0026 | -0.0026 | -0.0033 | -0.0023 | -0.0026 | -0.0019 |
| Getov | -0.2488 | -0.2611 | -0.2789 | -0.1663 | -0.1439 | -0.1518 | -0.2301 |
| phi | 0.0226 | 0.0216 | 0.0223 | 0.0070 | 0.0061 | 0.0060 | 0.0230 |
| TPSA | 0.0004 | 0.0000 | -0.0001 | 0.0007 | 0.0003 | 0.0003 | 0.0000 |
| UI | -0.2429 | -0.1967 | -0.1872 | -0.2530 | -0.1619 | -0.1767 | -0.1613 |
| Hy | 0.3055 | 0.2218 | 0.1983 | 0.4122 | 0.2497 | 0.2765 | 0.1543 |

**PARTIAL LEAST SQUARES REGRESSION RESULTS**

Table S4 – Summary of ANOVA and Fit Model Statistics from Partial Least Squares Regression (PLSR) Analysis

| **Molecular target** | **ANOVA Analysis** | | **Model Selection** | | | | |
| --- | --- | --- | --- | --- | --- | --- | --- |
|  | ***F*-value** | ***p*-value** | **no of components** | **X-variance** | **R^2^** | **PRESS** | **Predicted R^2^ (Q^2^)** |
| 6HEZ | 38.27 | <0.0001 | 2 | 0.8516 | 0.7847 | 18.84 | 0.6857 |
| 4BFT | 32.3 | <0.0001 | 2 | 0.8516 | 0.7541 | 19.04 | 0.6183 |
| 2FUM | 35.22 | <0.0001 | 2 | 0.8516 | 0.7703 | 17.90 | 0.6379 |
| 1ENY | 93.72 | <0.0001 | 2 | 0.8516 | 0.8992 | 9.06 | 0.8612 |
| 5V3X | 41.89 | <0.0001 | 2 | 0.8516 | 0.7996 | 10.87 | 0.6937 |
| 4UOG | 45.67 | <0.0001 | 2 | 0.8516 | 0.8131 | 10.50 | 0.7449 |
| 2WGE | 34.44 | <0.0001 | 2 | 0.8516 | 0.7663 | 8.83 | 0.6839 |

Table S5 – Summary of the significance of molecular descriptors to respective molecular targets in the PLSR Analysis

|  | **6HEZ** | **4BFT** | **2FUM** | **1ENY** | **5V3X** | **4U0G** | **2WGE** |
| --- | --- | --- | --- | --- | --- | --- | --- |
| Mol wt | **** | **** | **** | **** | **** | **** | **** |
| nhyd | ns | ns | ns | ** | * | * | ns |
| nhev | **** | **** | **** | **** | **** | **** | **** |
| noxy | ns | ns | * | ns | ns | ns | ns |
| nring | **** | **** | **** | **** | **** | **** | **** |
| nnitro | ns | * | * | ns | ns | ns | * |
| nrot | ns | ns | ns | ns | ns | ns | ns |
| ndonr | ** | * | * | **** | ** | ** | * |
| naccr | ns | ns | ns | * | ns | ns | ns |
| ndb | * | ** | ** | * | ns | ns | ** |
| LogP | ** | ns | ns | **** | ** | ** | ns |
| W | **** | **** | **** | **** | **** | **** | **** |
| Pol | **** | **** | **** | **** | **** | **** | **** |
| ISIZ | *** | ** | *** | **** | **** | **** | ** |
| TIAC | **** | **** | **** | **** | **** | **** | **** |
| Getov | * | ** | ** | * | ns | * | ** |
| phi | ns | ns | ns | ns | ns | ns | ns |
| TPSA | ns | ns | ns | ** | ns | ns | ns |
| UI | **** | *** | *** | **** | *** | **** | **** |
| Hy | *** | ** | ** | **** | *** | **** | ** |

Table S6 – Standardised Coefficients of molecular descriptors on the respective molecular targets in the PLSR analysis

| **MDs** | **6HEZ** | **4BFT** | **2FUM** | **1ENY** | **5V3X** | **4U0G** | **2WGE** |
| --- | --- | --- | --- | --- | --- | --- | --- |
| Constant | 0 | 0 | 0 | 0 | 0 | 0 | 0 |
| Mol wt | -0.0880 | -0.0856 | -0.0858 | -0.0942 | -0.0886 | -0.0893 | -0.0867 |
| nhyd | -0.0265 | -0.0333 | -0.0369 | -0.0274 | -0.0317 | -0.0319 | -0.0316 |
| nhev | -0.0976 | -0.0939 | -0.0936 | -0.1046 | -0.0975 | -0.0984 | -0.0953 |
| noxy | -0.0073 | -0.0167 | -0.0212 | -0.0067 | -0.0138 | -0.0138 | -0.0142 |
| nring | -0.1761 | -0.1577 | -0.1516 | -0.1901 | -0.1681 | -0.1697 | -0.1633 |
| nnitro | -0.0243 | -0.0297 | -0.0326 | -0.0253 | -0.0285 | -0.0287 | -0.0284 |
| nrot | -0.0129 | -0.0202 | -0.0238 | -0.0129 | -0.0181 | -0.0182 | -0.0183 |
| ndonr | 0.0760 | 0.0558 | 0.0473 | 0.0836 | 0.0644 | 0.0651 | 0.0615 |
| naccr | 0.0123 | 0.0006 | -0.0047 | 0.0146 | 0.0048 | 0.0049 | 0.0038 |
| ndb | -0.0794 | -0.0745 | -0.0733 | -0.0853 | -0.0781 | -0.0788 | -0.0761 |
| LogP | -0.1102 | -0.0871 | -0.0778 | -0.1204 | -0.0975 | -0.0985 | -0.0937 |
| W | -0.0759 | -0.0749 | -0.0756 | -0.0811 | -0.0771 | -0.0778 | -0.0755 |
| Pol | -0.0900 | -0.0874 | -0.0875 | -0.0963 | -0.0904 | -0.0912 | -0.0885 |
| ISIZ | -0.0563 | -0.0589 | -0.0610 | -0.0597 | -0.0594 | -0.0599 | -0.0585 |
| TIAC | -0.0722 | -0.0726 | -0.0737 | -0.0771 | -0.0742 | -0.0748 | -0.0728 |
| Getov | -0.1086 | -0.0995 | -0.0968 | -0.1169 | -0.1052 | -0.1061 | -0.1024 |
| phi | 0.0278 | 0.0141 | 0.0080 | 0.0313 | 0.0193 | 0.0196 | 0.0178 |
| TPSA | 0.0321 | 0.0174 | 0.0109 | 0.0360 | 0.0231 | 0.0234 | 0.0214 |
| UI | -0.1579 | -0.1321 | -0.1220 | -0.1717 | -0.1445 | -0.1459 | -0.1396 |
| Hy | 0.1493 | 0.1344 | 0.1294 | 0.1611 | 0.1430 | 0.1443 | 0.1389 |
